# Effectiveness of rehabilitation on pain and function in people affected by hemophilia

**DOI:** 10.1097/MD.0000000000027863

**Published:** 2021-12-17

**Authors:** Dalila Scaturro, Maria Grazia Benedetti, Giulia Lomonaco, Sofia Tomasello, Maria Grazia Giuseppina Farella, Giuseppina Passantino, Antonio Frizziero, Giulia Letizia Mauro

**Affiliations:** a Department of Surgical, Oncological and Stomatological Disciplines, University of Palermo Rehabilitation Unit, Paolo Giaccone Hospital, Palermo, Italy; b Physical Medicine and Rehabilitation Unit, University of Bologna, IRCCS-Istituto Ortopedico Rizzoli, Bologna, Italy; c Physical and Rehabilitation Medicine, Department of Medicine and Surgery General Hospital, University of Parma, Italy.

**Keywords:** arthritis, hemophilia, musculoskeletal rehabilitation, pain

## Abstract

**Introduction:**

Literature provides unclear evidence of the effectiveness of many physiotherapy interventions on pain intensity, quality of life, and functional ability in hemophilic patients, and suggests that rehabilitation programs should be focused on functional goals and the disability of patients.

**Aim:**

The aim of the present study is to present the outcome of a case series of patients with hemophilia in which a rehabilitation program has been carried out on the basis of a specific individual patient's functional impairment.

**Methods:**

Fifty-one patients were enrolled: 32 patients (Rehab Group) received a rehabilitative treatment, 19 patients for different reasons (living far from the hospital, family problems) did not receive rehabilitation (Control Group). The rehabilitation program was planned with respect to the emergent problems: musculoskeletal pain, joint range of motion limitation, muscle flexibility, walking difficulties, the appearance of hematomas/hemartro, and postural problems. All the patients were assessed at baseline (T0), after 1 month (T1), and after 3 months (T2) through visual analogic scale for musculoskeletal pain, the Hemophilia Joint Health Score for joint range of motion, the Functional Independence Score in Hemophilia for disability, and postural assessment by visual inspection.

**Results:**

A significant reduction of pain and improvement of Hemophilia Joint Health Score and Functional Independence Score in Hemophilia score was found in the Rehab Group along with the follow-up. Pain in the Control Group was lower with respect to the other group at baseline and significantly decreased after 1 month.

**Conclusion:**

A rehabilitation program focused on individual impairment of patients with hemophylia determined satisfying results in terms of pain control, functional, and disability improvement in 3 months follow-up.

## 1 Introduction

Hemophilia is a rare congenital coagulation disorder caused by the deficit or absence of clotting factors VIII (hemophilia a) and IX (hemophilia B).^[1]^ Due to the implications of the disease that causes marked involvement of the musculoskeletal system, a multidisciplinary approach is useful in hemophilic patients which, in addition to the hematologist, includes the involvement of professionals such as orthopedists, physiatrists, and physiotherapists.^[2]^ Between 35% and 50% of patients with hemophilia report living with chronic musculoskeletal pain^[3,4]^ with consequent limitations in mobility and independence, increased anxiety, restriction in activities of daily life, and reduced quality of life.^[5]^ Results of a recent survey highlight pain are mostly managed by the hemophilia specialists themselves, followed by a physiatrist and a physiotherapist. Currently, the main strategies indicated to manage musculoskeletal pain are pharmacological treatment, rehabilitation including physical therapy, and intra-articular injections.^[5]^ However, while evidence supports the role of physical exercise in promoting a reduction of perception of pain, and a better musculoskeletal function,^[6,7]^ there is unclear evidence of the effectiveness of many physiotherapy interventions on pain intensity, quality of life, and functional ability in these patients.^[3]^ In addition, it has been suggested that the rehabilitation program should be tailored to patients and be focused on the functional goal and on the slow achievement of autonomy which, once reached, must be maintained over time.^[8]^

The aim of the present study is to present the outcome of a case series of patients with hemophilia in which a tailored rehabilitation program has been carried out on the basis of the specific musculoskeletal impairment.

## 2 Methods

### 2.1 Ethical approval

This is a retrospective study, approved by the local Ethical committee (N. 3/2020, March 16, 2020). All the patients included in the study signed an informed consensus.

### 2.2 Patients

The entire cohort of hemophylic patients referred to the rehabilitation outpatient dedicated clinic in the years 2018 to 2019 were included in the study. The only exclusion criterion was age less than 6 years. Therefore, out of 52 patients, 1 child aged 2 years was excluded, and the other 51 patients were enrolled. Of these, 32 patients received, at different times, a rehabilitative treatment for the following reasons: musculoskeletal pain, joint range of motion limitation, muscle flexibility restriction, walking difficulties, the appearance of hematomas/hemartro, and postural problems. These patients were allocated to the Rehab Group. The remaining 19 patients were subjects who, despite needing to undergo rehabilitation, could not do it due to the distance from the hospital, or for family reasons. These patients received the same periodical assessment of the Rehab group, and were allocated to the Control Group. Symptomatic patients in this group were treated only with painkillers.

### 2.3 Rehabilitation program

The rehabilitation program was planned individually on patients with respect to the emergent problems assessed by clinical examination, with the following objectives: resolve/prevent hemarthro/hematoma, recover/maintain range of motion, recover/maintain muscle strength, prevent articular deformity, improve posture, and walking. The following activities were delivered: functional reeducation (restore joint range of motion depending on the anatomical site involved, muscle flexibility, and strength recovery), global postural reeducation, walking training, physical therapy (low-level laser therapy and kinesio-taping in the site of hematoma/hemarthro).

The rehabilitation program took place in 20 sessions (from Monday to Friday) lasting 60 minutes. Specifically, it included joint mobilization exercises, also in traction, and muscle stretching. Both methods are low-load maneuvers that allow to maintain or restore the tissue (muscles and tendons), promoting the proper joint movement. Muscle strength recovery was carried out through the initial execution of isotonic exercises, followed by low-load and high-repetition exercises. Achieved good muscle control and initial recovery, we proceeded on with the execution of isometric exercises, first with open and then with closed kinetic chain, followed by concentric and finally eccentric exercises.^[9]^

This was associated with the proprioceptive-education through the use of oscillating tablets, irregular surfaces, and visual biofeedback for the recovery of the execution of the specific gesture which, according to Wagner et al,^[10]^ prevents relapses. Hemarthrosis and hematomas can be considered the basis of the altered neuromuscular control provided by joint and muscle-tendon receptors. Furthermore, global postural re-education techniques were associated to improve the static and dynamic postural structure, and consequently the load distribution.

Finally, the various phases of the gait were analyzed and, based on the errors found, exercises aimed at improving gait patterns and patient education to recognize compensations and errors were performed, so they could correct themselves autonomously during the day.

All hemophilic patients continued with prophylaxis as per the protocol prescribed by the hematologist. The intake of COX-2 inhibitors and/or analgesics was forbidden for the entire duration of physiotherapy to eliminate confounding factors in the outcome of the study.

### 2.4 Outcome measures

All the patients in both Groups were assessed at baseline (T0), after 1 month (T1), and after 3 months (T2). For the Rehab Group, T0 coincided with the pre-rehabilitation assessment and T1 the assessment at the end of the rehabilitation program.

The following measures were used for outcome assessment: visual analogic scale (VAS) for joint pain (the maximum pain reported in the week before assessment, independently from the joint was collected), the Hemophilia Joint Health Score (HJHS),^[11]^ and the Functional Independence Score in Hemophilia (FISH).^[12]^ The postural assessment was carried out in static posture, observing the alignment of the patient in different planes, noting the position of the cervical and thoracic spine, the relationship of the thorax and the pelvis, and the position of the shoulder girdle.

The HJHS measures joint health, in the domain of body structure and function (ie, impairment), of the joints most commonly affected by bleeding in hemophilia: the knees, ankles, and elbows. It provides a maximum score of 124 points (a higher score is worse).

FISH is a performance-based assessment tool to objectively measure an individual's functional ability. It includes the assessment of 8 activities: eating, grooming, dressing, chair transfer, squatting, walking, step climbing, and running. Each activity is graded from 1 to 4 according to the amount of assistance required to perform the activity. The maximum score is 32 (the greatest autonomy).

### 2.5 Statistical analysis

All continuous data were summarized in terms of mean ± standard deviation. Analysis of variance for repeated measures with post hoc pairwise comparisons by Sidak test was used to analyze VAS, HJHS along with the follow-up in the Rehab Group. Friedman non-parametric test followed by Wilcoxon post hoc pairwise test corrected for multiple comparisons with Bonferroni was used in the Control Group, due to the small number of patients.

The statistical analysis was performed using the statistical package for social sciences (SPSS), software version 15.0 (SPSS Inc., Chicago, USA) by a statistical consultant from our institute.

## 3 Results

For the Rehab Group, the mean age at the time of admission to the clinic was 34.4 years old (SD 14.7, range 17–62), all males. The diagnosis was as follows: 19 patients had severe hemophilia A, 5 had mild-moderate hemophilia A, 4 had severe hemophilia B, 1 had mild hemophilia B, and 3 other diagnosis (Von Willebrand, deficit factor II and deficit factor V). All patients were on specific drug therapy. Nine patients were students, 5 were office workers, 5 were unemployed, 3 were workmen, 8 were professionals in various fields, and 2 were retired. All the patients have suffered from an arthropathy or an emarthro at different sites: elbows, knees, and ankles. Fourteen patients played regularly some sport activity, 18 patients none.

For the Control Group, mean age at the time of admission to the clinic was 24.4 years old (SD 16.5, range 7–54), all males. The diagnosis was as follows: 8 patients had severe hemophilia A and 11 had mild-moderate hemophilia A. All patients were on specific drug therapy. Eleven patients were students, 2 were office workers, 4 were workmen, 1 was unemployed, and 1 was military. Eleven patients have suffered from an arthropathy or an hemarthro at different sites: elbows, knees, and ankles. Eleven patients played regularly some sport activity and 8 patients none. General data of patients are reported in Table 1.

**Table 1:** General data.

	Rehab Group (N. 32)	Control Group (N. 19)
Age	34.4 (14.7)	24.4 (16.5)
BMI		
Hemophilia		
A severe	19	8
A mild-moderate	5	11
B severe	4	
B Mild	1	
Others	3	
Sport	14 (yes) 18 (no)	11 (yes) 7 (no)
Occupation		
Students	9	11
Office workers	5	2
Workmen	3	4
Military		1
Professionals	8	
Unemployed	5	1
Retired	2	
Arthropathy		
Ankle right/left	14	6
Knee right/left	13	4
Elbow	8	2

The 2 groups differed for age since the Rehab Group was older as a mean age (34.4 ± 14.7) with respect to the Control Group (24.2 ± 16.5), and also with a greater number of patients with severe hemophilia A and arthropathy (19 in the Rehab Group vs 8 in the No Reha Group).

Results of outcome assessment in the 2 groups in terms of pain control, functional ability, and disability along the follow-up are reported in Table 2.

**Table 2:** Outcome measures.

Groups	Variable	Mean	SD	ANOVA repeated measures/^∗^Friedman non-parametric test	Post-hoc pairwise comparisons with Sidak Test /^∗^Wilcoxon post hoc pairwise test		Sig. *P*
Rehab (n.32)	VAS T0	4.8	2.0	*P* < .0005	T0	T1	<.0001
	VAS T1	3.4	1.9			T2	<.0002
	VAS T2	2.9	2.1		T1	T2	.035
Rehab	HJHS TO	27.5	24.3	*P* = .01	T0	T1	.01
	HJHS T1	23.9	21.8			T2	.039
	HJHS T2	23.8	23.5		T1	T2	.999
Rehab	FISH TO	26.2	5.8	*P* = .002	T0	T1	.003
	FISH T1	28.0	4.2			T2	.002
	FISH T2	28.3	4.1		T1	T2	.656
No Rehab (n.19)	VAS T0	3.0	2.6	^∗^*P* = .02	T0	T1	^∗^.023
	VAS T1	2.6	2.4			T2	^∗^.07
	VAS T2	2.5	2.4		T1	T2	^∗^.999
No Rehab	HJHS TO	10.5	12.1	^∗^*P* = .716	T0	T1	^∗^.999
	HJHS T1	10.0	11.3			T2	^∗^.999
	HJHS T2	9.8	10.9		T1	T2	^∗^.999
No Rehab	FISH TO	29.2	4.4	^∗^*P* = .117	T0	T1	^∗^.094
	FISH T1	30.5	3.9			T2	^∗^.094
	FISH T2	30.6	3.4		T1	T2	^∗^.999

ANOVA = analysis of variance, FISH = functional independence score in hemophilia, HJHS = Hemophilia Joint Health Score, T0 = baseline, T1 = after 1 month, T2 = after 3 months, VAS = visual analogic scale for pain.

Pain in the Rehab Group significantly decreased after rehabilitation (from 4.8 ± 2 to 3.4 ± 1.9, *P* < .0001) and a further significant decrease vs evident at 3 months follow up (2.9 ± 2.1, *P* < .0002). Functional ability as measured by means of the FISH also significantly increased after rehabilitation (from 26.2 ± 5.8 to 28 ± 4.2, *P* = .003) and was stable at 3 months follow up (28.3 ± 4.1). HJHS score also significantly improved after rehabilitation (from 27.5 ± 24.3 to 23.9 ± 21.8, *P* = .01) and remained stable at 3 months follow up (23.8 ± 23.5). Pain in the Control Group was lower with respect to the other Group at baseline (3 ± 2.6) and significantly decreased after 1 month (2.6 ± 2.4) (*P* = .023). No other changes were evident along with the follow-up both for FISH and HJHS in this group.

## 4 Discussion

The availability of medical treatment with coagulation factors for patients with hemophilia has completely changed the rehabilitation approach to these patients.^[7,13]^ If in the past motor activities and exercise were often inhibited, today there is wide evidence that therapeutic exercise is a pillar of musculoskeletal problems treatment.^[13]^ In particular physical therapy protocols are able to break the vicious cycle of inactivity, kinesiophobia, and chronic musculoskeletal pain related to repeated hemartroses, muscle atrophy, and joint instability, improving social and emotional well-being.^[6,14]^ Despite replacement therapy, in fact, patients can continue to have bleeding in joints and muscles with consequent chronic arthropathy. For these reasons the rehabilitation treatment of hemophylic patients remains complex, and guidelines for rehabilitation prescription based on scientific evidence are required. Many reviews searched for current evidence of the effectiveness of physiotherapy interventions on musculoskeletal pain and function.^[3,6,7,15]^ All authors agree on the multidisciplinary approach to patients with hemophylia, confirming the need for physiotherapy and its safety with respect to bleeding related to exercise,^[7]^ but unclear conclusions are available with respect to the best intervention to recommend.^[3]^ Most of the authors reported benefits by physiotherapy programs based on the recovery of flexibility, strength, balance, proprioception, and aerobic capacity both through land-based and aquatic exercise^[8,10,14,16]^ as well by physical modalities (transcutaneous electrical nerve stimulation laser, ultrasounds, neuromuscular electrical stimulation, electromagnetic fields, and kinesiotaping)^[5,17,18]^ and joint mobilizations.^[15,16,19]^

However, if we consider the increasing interest in optimizing profilaxyx treatment on an individualized patient pharmacokinetic profile, similarly, in our opinion, it is very important that also the rehabilitation program could be planned on the basis of individual clinical-functional features of the patients, and not proposed with standardized protocols. Each patient in fact has its own manifestation of disease, with different sites involved, and joints and muscles affected by arthropathies and contractures of different levels of severity. That is why, the individual assessment in terms of impairment, disability, and participation is of paramount relevance to customize the rehabilitation program to the specific needs of patients.^[13,20]^

In the present study patients with chronic pain and musculoskeletal complications due to hemophilia were assessed through clinical evaluation for pain (VAS), joint impairment (HJHS), postural alignment, walking abnormalities, and functional ability (FISH). According to the individual features of the patient a rehabilitation program was planned. Our findings demonstrated an improvement of all the scores we used as an outcome measure, up to 3 months after treatment, thus supporting the effectiveness of the program undertaken compared to the Control Group in which only pain improved, without any significant change in functional limitation and disability.

### 4.1 Limitations of the study

As a limitation of the study, patients in the intervention group were older than controls and with much more severe forms of hemophilia, joint involvement, the intensity of pain, and disability, but just for these reasons, they had been deemed worthy of a specific rehabilitation.

In the group of patients who did not carry out a rehabilitation program and that we used as a Control Group, no change was found, except a slight reduction of pain, probably related to the medicaments for pain control. Of course, the approach used in the present study has the limit that it is not reproducible in other studies because the rehabilitative intervention was based on a problem-solving approach through a rehabilitation program addressed to the specific problems of the single patient. Furthermore, the allocation of patients in the 2 groups was based on the delivery or not of rehabilitation, without randomization. Methodologically more robust studies, in particular randomized controlled studies, are desirable to demonstrate the rehabilitation effectiveness in these patients.

## 5 Conclusions

In conclusion, a rehabilitation program based on individual impairment for patients with hemophylia was demonstrated to provide satisfying results in terms of pain control, functional, and disability improvement at 3 months follow-up. An open problem remains the consolidation of the results obtained and the prevention of deterioration of the musculoskeletal condition. Cycles of rehabilitation planned on specific emerging clinical problems can be periodically delivered, but it is very important that patients maintain a correct lifestyle, participate in fitness, or adapted physical activity programs to guarantee long-term physical wellness.

## Author contributions

**Conceptualization:** Giulia Letizia Mauro, Maria Grazia Benedetti.

**Data curation:** Dalila Scaturro, Sofia Tomasello.

**Data Collection:** Giuseppina Passantino.

**Investigation:** Giulia Lomonaco, Maria Grazia Giuseppina Farella.

**Methodology:** Dalila Scaturro, Antonio Frizziero, Maria Grazia Benedetti

**Supervision:** Giulia Letizia Mauro, Maria Grazia Benedetti.

**Writing – original draft:** Dalila Scaturro, Giulia Letizia Mauro, Antonio Frizziero, Maria Grazia Benedetti

**Writing – review & editing:** Antonio Frizziero, Maria Grazia Benedetti
