# Effectiveness of rehabilitation on pain and function in people affected by hemophilia: Erratum

**DOI:** 10.1097/MD.0000000000028715

**Published:** 2022-01-28

In the article, “Effectiveness of rehabilitation on pain and function in people affected by hemophilia”,^[1]^ which appears in Volume 100, Issue 50 of *Medicine*, Giuseppina Passantino has been added to the author list.
